# Microbial rRNA gene expression and co‐occurrence profiles associate with biokinetics and elemental composition in full‐scale anaerobic digesters

**DOI:** 10.1111/1751-7915.13264

**Published:** 2018-04-06

**Authors:** Ryan M. Ziels, Bo H. Svensson, Carina Sundberg, Madeleine Larsson, Anna Karlsson, Sepehr Shakeri Yekta

**Affiliations:** ^1^ Department of Civil Engineering The University of British Columbia Vancouver BC Canada; ^2^ Department of Thematic Studies‐Environmental Change Linköping University Linköping Sweden; ^3^ Biogas Research Center Linköping University Linköping Sweden; ^4^ Scandinavian Biogas Fuels AB Stockholm Sweden

## Abstract

This study examined whether the abundance and expression of microbial 16S rRNA genes were associated with elemental concentrations and substrate conversion biokinetics in 20 full‐scale anaerobic digesters, including seven municipal sewage sludge (SS) digesters and 13 industrial codigesters. SS digester contents had higher methane production rates from acetate, propionate and phenyl acetate compared to industrial codigesters. SS digesters and industrial codigesters were distinctly clustered based on their elemental concentrations, with higher concentrations of NH_3_‐N, Cl, K and Na observed in codigesters. Amplicon sequencing of 16S rRNA genes and reverse‐transcribed 16S rRNA revealed divergent grouping of microbial communities between mesophilic SS digesters, mesophilic codigesters and thermophilic digesters. Higher intradigester distances between *Archaea* 16S rRNA and rRNA gene profiles were observed in mesophilic codigesters, which also had the lowest acetate utilization biokinetics. Constrained ordination showed that microbial rRNA and rRNA gene profiles were significantly associated with maximum methane production rates from acetate, propionate, oleate and phenyl acetate, as well as concentrations of NH_3_‐N, Fe, S, Mo and Ni. A co‐occurrence network of rRNA gene expression confirmed the three main clusters of anaerobic digester communities based on active populations. Syntrophic and methanogenic taxa were highly represented within the subnetworks, indicating that obligate energy‐sharing partnerships play critical roles in stabilizing the digester microbiome. Overall, these results provide new evidence showing that different feed substrates associate with different micronutrient compositions in anaerobic digesters, which in turn may influence microbial abundance, activity and function.

## Introduction

Anaerobic digestion is a microbial process that produces renewable methane from various organic waste streams and is expanding globally due to a growing interest in sustainable energy and nutrient recovery (McCarty *et al*., 2011; Batstone and Virdis, 2014). Efficient and stable conversion of organic matter into methane relies on a series of interdependent relationships between fermenters, acidogens, acetogenic syntrophs and methanogenic archaea (Stams, 1994; Briones and Raskin, 2003). Due to the large microbial biodiversity present in anaerobic digesters (Nelson *et al*., 2011; Sundberg *et al*., 2013) and the large portion of uncultured species in such systems (Li *et al*., 2009), culture‐independent molecular tools have been critical in advancing our understanding of microbial communities within anaerobic digesters (Rivière *et al*., 2009; Werner *et al*., 2011; Vanwonterghem *et al*., 2014, 2016; De Vrieze *et al*., 2015, 2016a; Hao *et al*., 2016). Despite these advancements, anaerobic digesters are still typically operated based on empirical knowledge of process parameters, owing to a general lack of understanding regarding relationships between microbial community structure and digester function in terms of process biokinetics (Carballa *et al*., 2015). While some recent studies have found correlations between specific population abundances and substrate conversion biokinetics (Venkiteshwaran *et al*., 2017; Ziels *et al*., 2017), these studies focused on the presence of DNA, which may provide little information about the *active* portion of the microbial community. Discrepancies have been observed between DNA‐based abundance and RNA‐ or protein‐based expression of methanogen populations in anaerobic digesters (Zakrzewski *et al*., 2012), which could be attributed to their high activity but low biomass yields (Smith *et al*., 2015) or conversely due to changes in their RNA/protein expression in response to environmental perturbations (De Vrieze *et al*., 2016b). For instance, changes in mRNA expression of the methyl‐coenzyme M reductase gene (*mcrA*) involved in methanogenesis were observed along with decreased methane production rates at high ammonia loadings, whereas DNA levels of the *mcrA* gene remained stable (Zhang *et al*., 2014a). While changes in the expression of ribosomal RNA (rRNA) may not always reflect changes in microbial activity over short temporal scales (e.g. 1–3 days) (Flärdh *et al*., 1992; Wagner *et al*., 1995), rRNA gene expression levels have been related to microbial activity and growth rates within bioreactors under steady‐state conditions (Poulsen *et al*., 1993). Comparing the rRNA (RNA) and rRNA gene (DNA) profiles of an anaerobic digester microbial community may highlight active populations more so than DNA‐only based approaches, due to the shorter half‐life of rRNA and constant turnover of digester contents by the solids retention time.

Many factors can impact the digester efficiency as well as the microbial community structure, such as temperature (De Vrieze *et al*., 2015; Westerholm *et al*., 2015), the hydraulic and solids retention times (Lee *et al*., 2011; Hao *et al*., 2016), salinity (De Vrieze *et al*., 2016b), free ammonia (Westerholm *et al*., 2015), feeding regime (Conklin *et al*., 2006; De Vrieze *et al*., 2013; Mulat *et al*., 2016; Ziels *et al*., 2017) and micronutrients (Karlsson *et al*., 2012). Among these important parameters, macro‐ and micronutrient levels are gaining attention because they are necessary for many biological and enzyme‐mediated processes in methanogenic communities, and they can be controlled via supplementation (Demirel and Scherer, 2011). A vast body of research has examined the effect of nutrient supplementation on the performance of the anaerobic digestion process (Worm *et al*., 2009; Banks *et al*., 2012; Karlsson *et al*., 2012; Williams *et al*., 2013; Schmidt *et al*., 2014; Zhang *et al*., 2015; Moestedt *et al*., 2016), highlighting that many anaerobic digesters may have suboptimal organic matter conversion and methane production due to deficiencies in one or multiple essential elements.

Our previous study of 21 full‐scale anaerobic digesters in Sweden demonstrated that different feed substrate types were associated with different methanogenic pathways and community structure (Sundberg *et al*., 2013). We suggested that differences in the elemental compositions of the feed substrates may be influential to establishing different microbial activities and functional performance of the digesters. However, there have been no studies examining the association of macro‐ and micronutrient levels with process biokinetics and the activities and abundance of microbial populations in full‐scale anaerobic digestion systems.

The objective of this research was to elucidate relationships between the activity of microbial populations and elemental concentrations and biokinetics within a diverse set of 20 full‐scale anaerobic digesters, including municipal sewage sludge (SS) digesters and industrial codigesters (Table 1). The digester *Bacteria* and *Archaea* community profiles were examined through amplicon sequencing of 16S rRNA genes and reverse‐transcribed 16S rRNA, and the concentrations of 21 biologically relevant elements were measured in the digester contents. Additionally, the biokinetics of methane production from acetate, propionate, phenyl acetate and oleate were assessed in batch assays with the full‐scale digester contents to infer their capacity to degrade critical intermediates in the methanogenic food chain. We examined potential correlations between 16S rRNA and rRNA gene profiles and elemental concentrations as well as biokinetics to elucidate whether different substrate types create distinct ecological conditions that drive differences in microbial activity and function within the full‐scale digesters.

**Table 1:** Operational parameters of the reactors including feed substrate type, temperature, organic loading rate (OLR), free ammonia and hydraulic retention time (HRT)

Reactor	Temp. (°C)	Substrate (% volume fraction of total substrate)	pH	Free Ammonia (mg NH_3_‐N l^−1^)	OLR (kg VS m^−3^ day^−1^)	HRT (days)
CD1	38	Wheat stillage (47%), grain (37%) and water (14%)	7.5	204	3.3	55
CD10	38	OFMSW (77%), fat (12%) and silage (11%)	7.7	162	2.2	28
CD11ap	38	SHW in majority	8.0	558	3.7	55
CD11 bp	38	SHW in majority	8.0	541	3.7	55
CD2T	53	OFMSW (62%), dry fodder (13%) and SHW (9%)	7.9	424	2.4	25
CD3T	52	SHW (45%), manure (cow) (29%) and whey (14%)^a^	7.9	751	3.9	20
CD4ap	37	SHW (45%), manure (pig and cow) (32%) and OFMSW (20%)^a^	7.7	178	3.4	25
CD6ap	38	Manure (pig and cow) (67%) and SHW/OFMSW (32%)^b^	7.9	289	3.6	26
CD6 bp	38	Manure (pig and cow) (67%) and SHW/OFMSW (32%)^b^	7.9	261	3.6	26
CD7a	37	OFMSW (57%), FIW (21%) and SHW (12%)2)^c^	7.6	123	2.5	35
CD7b	37	OFMSW (57%), FIW (21%) and SHW (12%)2)^c^	7.3	49	3.6	24
CD8Tap	53	OFMSW (95%) and fat (5%)	7.7	176	3.2	20
CD9T	55	OFMSW (85%) and SHW (15%)	8.2	803	1.8	‐
SS1as	37	FIW (58%) and PS/WAS (42%)	7.1	13	3	39
SS1bs	37	Sludge from SS1as	7.3	20	1.1	39
SS2T	52	PS/WAS	7.5	112	2.5	12
SS3a	37	PS	7.1	16	2.7	14
SS3b	37	PS (54%) and WAS (46%)	7.2	26	2.4	10
SS4ap	37	WAS (55%), PS (42%), sludge from external source (3%)	7.6	39	2.4	18
SS5	38	PS	7.3	26	–	50

FIW, food industry waste; OFMSW, organic fraction municipal solid waste; PS, primary sewage sludge; SHW, slaughterhouse waste; WAS, waste‐activated sludge.

**a**. 2–6% fat is included in the remaining substrate.

**b**. 1–1.5% iron‐rich sludge is included in the remaining substrate.

**c**. 8% pig manure is included in the remaining substrate.

## Results and discussion

### Elemental composition in full‐scale anaerobic digesters

**Figure 1:**
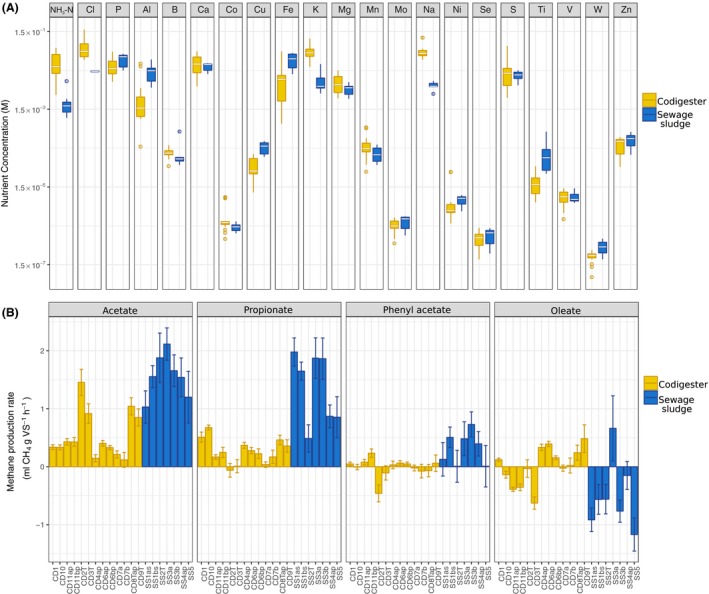
A. Box plot illustrating average concentrations of the measured elements in the codigesters and sludge digesters. B. Maximum methane production rates (*R*_m_) from batch assays with acetate, propionate, phenyl acetate and oleate with all digester contents determined via fitting Gompertz models to methane production curves. *R*_m_ values refer to standard temperature and pressure. Error bars indicate the standard errors for *R*_m_ using the nonlinear regression analysis.

We assessed the concentrations of 21 elements within 20 full‐scale anaerobic digesters (Table 1) with concurrent analysis of biokinetics and microbial rRNA and rRNA gene profiles. NMDS analysis based on Bray–Curtis distances of elemental concentrations in the digestate samples demonstrated that the SS digesters were grouped separately from codigesters (Fig. S1). The dissimilarity in elemental profiles in codigesters versus SS digesters was confirmed by ANOSIM (*R*_ANOSIM_ = 0.75, *P < *0.001). The SS digesters had higher Cu, Al, Ti, W, Ni and Fe concentrations (Fig. 1A) than codigesters. The higher Cu concentration in SS digesters may be related to leaching from drinking water and wastewater collection pipes (Boulay and Edwards, 2001; Sörme and Lagerkvist, 2002), while higher Al and Fe levels could be due to their common supplementation for phosphate removal prior to anaerobic digestion at wastewater treatment plants (Wilfert *et al*., 2015). Trace amounts of Ni present in chemical stocks used for phosphate removal have been shown to cause elevated Ni levels in sewage sludge (Sörme and Lagerkvist, 2002). Codigesters typically had higher concentrations of NH_3_‐N, Cl, K and Na (Fig. 1A). Many of the codigesters received slaughterhouse waste and food waste (Table 1), which are protein‐rich substrates that could lead to high digester free ammonia levels (Palatsi *et al*., 2011). Within the overall cluster of codigester elemental profiles, CD1, CD11ap and CD11 bp were closely grouped (Fig. S1). These codigesters are operated by a private company and are supplemented with a proprietary nutrient additive containing mainly Fe, Co and Ni (data not shown). Overall, these results demonstrate that municipal sludge digesters and codigesters have distinct elemental compositions, which may be attributed to differences in influent feedstocks and/or differences in chemical supplementation (e.g. salts for P removal).

### Degradation kinetics of key substrates by the full‐scale digester contents

Batch assays were conducted over 280 h with digestate sampled from the 20 full‐scale digesters to measure their degradation kinetics for acetate, propionate, phenyl acetate and oleate (Fig. 1B). The purpose of the batch incubations was to capture the kinetics of substrate degradation by the microbial communities, not to monitor the time until total substrate degradation. Background VFA levels in the full‐scale digester contents were below 20 mM (Table S1) and, thus, likely did not impact maximum observed rates in the batch assays. Maximum methane production rates (*R*_m_) from acetate and propionate were higher with contents from the SS digesters compared to the codigesters (*P *<* *0.01, Welch's *t*‐test) (Fig. 1B). Yet, acetate conversion in the codigesters was faster under thermophilic conditions than under mesophilic conditions (*P *<* *0.01, Welch's *t*‐test) (Fig. 1B). Maximum methane production rates in the batch assays supplemented with phenyl acetate were lower than in the unsupplemented control assays for several codigesters (Table S2), resulting in negative net substrate conversion rates (Fig. 1B). However, the rates of phenyl acetate degradation were slightly positive in the SS reactors, except for SS1as, SS2T and SS5. Phenyl acetate was previously shown to stimulate methanogenic rates in anaerobic digesters fed with swine manure, but had inhibitory effects in digesters fed with kitchen food waste (Hecht and Griehl, 2009). Thus, the methane production rate from phenyl acetate could depend on the microbial community structure, as the anaerobic degradation of phenyl acetate was hypothesized to rely on syntrophic interactions (Glissmann *et al*., 2005). Oleate addition resulted in lower methane production rates than in unsupplemented controls for assays with SS digester contents, except for SS3a, while five codigesters had slightly positive net oleate conversion rates (Fig. 1B). NMDS analysis showed that the biokinetic profiles of the SS digesters and the codigesters were distinctly clustered (Fig. S1), and the significance of the clustering was confirmed by ANOSIM (*R*_ANOSIM_ = 0.8, *P < *0.001). Overall, these results demonstrate that the biokinetic characteristics of full‐scale digester communities were distinct among codigesters and SS digesters, with SS digesters having higher acetate, propionate and phenyl acetate conversion rates and lower oleate conversion rates in comparison with codigesters.

### Microbial community structure and activity in full‐scale digesters

**Figure 2:**
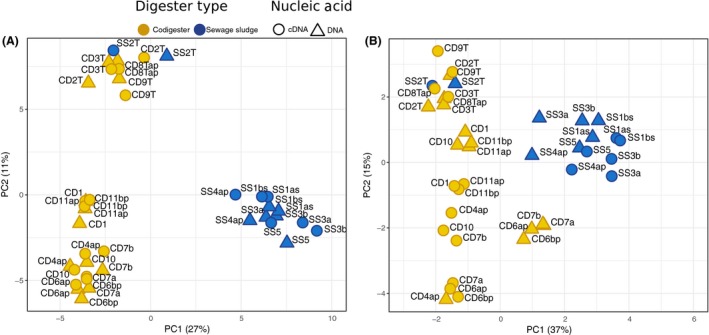
Principal components analysis (PCA) plots of (A) *Bacteria* and (B) *Archaea* OTU read counts after variance stabilizing transformation with DESeq2 (Love *et al*., 2014). Points are shown for both rRNA gene (DNA) and rRNA (RNA) samples for each digester. Values in parentheses represent the fraction of the total variance explained by each PC axis.

The microbial community structure and activity of the full‐scale digester contents were assessed with parallel amplicon sequencing of both 16S rRNA and rRNA genes. Principal components analysis (PCA) of OTU read counts in the rRNA and rRNA gene pools of the 20 full‐scale digesters showed that *Bacteria* and *Archaea* community structures were clustered into three main groups: thermophilic digesters, mesophilic SS digesters and mesophilic codigesters (Fig. 2). This finding confirms previous observations that temperature and substrate type are major factors that influence the microbial community structure in anaerobic digestion (Sundberg *et al*., 2013; Zhang *et al*., 2014b; De Vrieze *et al*., 2015). However, this work is the first to demonstrate such clustering based on active communities via reverse‐transcribed 16S rRNA profiles of full‐scale anaerobic digesters.

The distances between intradigester *Bacteria* community DNA and RNA profiles were smaller than for *Archaea* (Fig. 2A), meaning that the bacterial DNA profiles were more reflective of their activity (RNA). The largest intradigester DNA and RNA distances were observed for *Archaea* community profiles within the mesophilic codigester samples, including CD1, CD4ap, CD6ap, CD6 bp, CD7a, CD7b, CD10, CD11ap and CD11 bp (Fig. 2B). Notably, these codigesters had the lowest acetate conversion rates of all digesters tested (Fig. 1A). Large deviations between archaeal RNA and DNA gene profiles for 16S rRNA were previously associated with stressful conditions during anaerobic digestion caused by high salinity (De Vrieze *et al*., 2016b) and high LCFA levels (Amha *et al*., 2017). If larger archaeal 16S rRNA and rRNA gene distances are indicative of stressful conditions, this would suggest that the full‐scale codigesters had higher stress levels than SS digesters. This supports the hypothesis that rRNA signatures may be representative of deterministic processes under stable digester conditions, while under periods of process perturbation, the rRNA signatures may reflect stochastic processes (De Vrieze *et al*., 2016b). The *Archaea* communities in the mesophilic codigesters in this study may have had divergent rRNA and rRNA gene profiles due to stress induced from high ammonia levels (Table 1), or possibly from variable feed substrate compositions during codigestion operations. For instance, De Francisci *et al*. (2015) showed that a changing feed composition during codigestion impacted the microbial community structure based on rRNA genes. Yet, to the best of our knowledge, the impact of changing feed substrate composition on 16S rRNA gene expression has not been elucidated. Further time‐series analysis is needed to verify whether *Archaea* rRNA and rRNA gene distances can be predictive of lower digester biokinetics in full‐scale systems, and whether a changing feed composition during codigestion impacts *Archaea* 16S rRNA gene expression.

**Figure 3:**
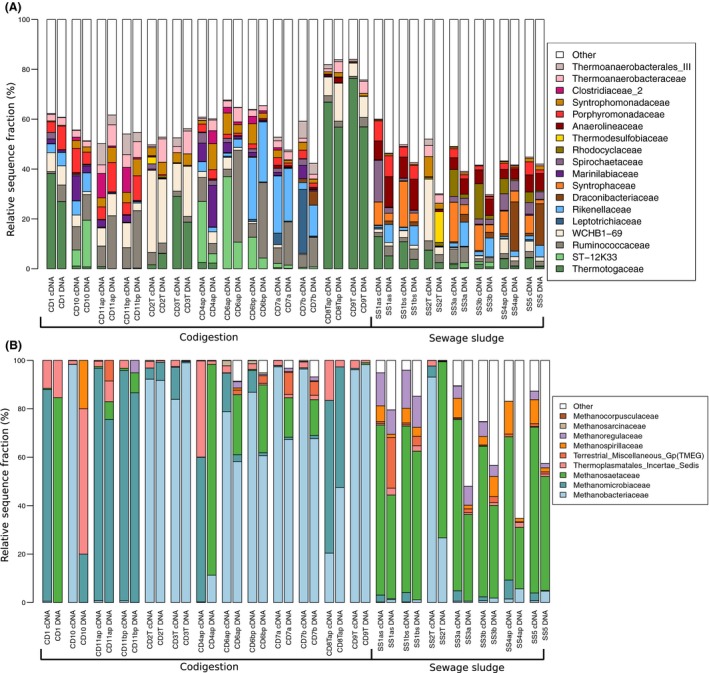
Relative sequence fractions of (A) the 18 most abundant *Bacteria* families and (B) the 10 most abundant *Archaea* families, in the 16S rRNA and rRNA gene profiles of all full‐scale digesters. rRNA fractions are denoted ‘cDNA’, and rRNA gene fractions are denoted ‘DNA’. Read fractions not taxonomically classified within the most abundant families are included as ‘other’. WCHB1‐69 and ST‐12K33 are uncultured groups under the order *Sphingobacteriales*.

The taxonomic profiles of the *Bacteria* communities also differed between the SS digesters and codigesters, corresponding to the observed OTU clustering patterns between the digester groups. Over 85% of bacterial reads were attributed to 12 phyla (Fig. S2). The SS digesters had higher fractions of *Syntrophaceae* in both the rRNA and rRNA gene fractions in comparison with the codigesters (Fig. 3A). The presence of *Syntrophaceae* in the SS digesters was mostly attributed to the propionate‐oxidizing genus of *Smithella* (Fig. S3). The *Candidatus* Cloacamonas genus was also detected in higher abundance and activity levels in the SS digesters relative to the codigesters (Fig. S3). This genus includes potential amino acid‐ and propionate‐degrading syntrophs (Pelletier *et al*., 2008; Sieber *et al*., 2012) and could have been enriched in the SS digesters due to hydrolysis of cellular proteins in the feed primary and waste‐activated sludge (PS+WAS). Conversely, the *Syntrophomonadaceae* family typically had higher rRNA and rRNA gene sequence fractions in the codigesters relative to the SS digesters, except for the thermophilic SS2T digester (Fig. 3A). *Syntrophomonadaceae* sequences consisted mostly of the *Syntrophomonas* genus (Fig. S3), which includes short‐ and long‐chain fatty acid β‐oxidizing species (Sousa *et al*., 2009). *Syntrophomonas* has also been purported to belong to the core community within anaerobic digesters (De Vrieze *et al*., 2016a; Treu *et al*., 2016), which is corroborated by its presence in 95% of the digester rRNA samples in this study (19 of 20). Thermophilic codigesters generally had higher bacterial rRNA and rRNA gene sequence fractions of the family *Thermotogaceae,* which comprised over 80% of the rRNA reads in CD9T (Fig. 3A) and was mainly comprised of the genus *Defluviitoga* (Fig. S3). *Defluviitoga* was also the dominant genus in the rRNA and rRNA gene profiles in CD1, comprising 38% and 27% of bacterial reads respectively (Fig. S3). Genome reconstruction of a *Defluviitoga* spp. indicated it is a hydrolytic fermenting organism (Maus *et al*., 2016a). *Defluviitoga* spp. have been detected at high levels in other thermophilic digesters (Maus *et al*., 2016b) indicating its potential significance to methane production at high temperatures. Notably, genera harbouring known syntrophic acetate‐oxidizing (SAO) bacteria of *Tepidanaerobacter* and *Syntrophaceticus* (Westerholm *et al*., 2010, 2011) were detected at higher rRNA and rRNA gene fractions in the codigesters compared to SS digesters, again with the exception of the thermophilic SS2T digester (Fig. S3). The presence and activity of SAO in codigesters but not SS digesters were likely attributed to the higher level of ammonia–nitrogen in the codigesters (Fig. 1A), which is considered a driving factor for SAO in anaerobic digestion (Schnürer and Nordberg, 2008). This result also aligns with the potentially higher stress in the mesophilic codigester *Archaea* populations, as indicated by their large intradigester rRNA and rRNA gene distances (Fig. 2B). The SAO genus *Tepidanaerobacter* often had higher rRNA fractions relative to its rRNA gene fraction, indicating that it might have been highly active but slow growing.

The presence of SAO bacteria in codigesters corresponded with an absence of aceticlastic methanogens from their rRNA sequence pools (Fig. 3B). *Methanosaetaceae* was the primary aceticlastic methanogen family detected at high abundance in the *Archaea* rRNA and rRNA gene sequences of the mesophilic SS digesters, whereas *Methanosaetaceae* was only detected at a maximum 3% read fraction in the codigester rRNA samples (Fig. 3B). *Methanosaeta* is an obligate aceticlast with a higher affinity for acetate than the other aceticlastic genus of *Methanosarcina* (Conklin *et al*., 2006). While we have previously reported the dominance of *Methanosaeta* rRNA genes in digesters treating municipal sludge (Sundberg *et al*., 2013), the high rRNA gene expression of *Methanosaeta* in SS digesters found in this study (Fig. S3) shows that aceticlastic methanogenesis is likely the main acetate utilization pathway in such systems. The observation that *Methanosaeta* rRNA gene expression was low in thermophilic SS digesters and codigesters, even though it was present based on rRNA genes, suggests that rRNA profiling may accurately capture stochastic processes in methanogenic communities due to suboptimal growth conditions, as was suggested by De Vrieze *et al*. (2016b) during high salinity exposure. The hydrogenotrophic genus of *Methanospirillum* was also detected in the mesophilic SS digester *Archaea* rRNA and rRNA gene libraries, but was not detected in the rRNA sequences of the codigesters nor the thermophilic SS2T, suggesting that it was inactive in those systems (Fig. 3B, S3). In contrast, the codigester rRNA libraries were dominated by the hydrogenotrophic families of *Methanomicrobiaceae* and *Methanobacteriaceae* (Fig. 3B), which were mostly comprised of the genera *Methanoculleus* and *Methanothermobacter*, respectively (Fig. S3). *Methanoculleus* was often dominant in the mesophilic codigesters, whereas *Methanothermobacter* was prevalent in thermophilic codigesters and SS2T. Both *Methanoculleus* and *Methanothermobacter* are known partners of SAO bacteria at high free ammonia levels (Schnürer *et al*., 1999; Hattori *et al*., 2000; Manzoor *et al*., 2016). Often the *Methanoculleus* rRNA read fraction was larger than its rRNA gene fraction (Fig. S3), suggesting that members of this genus were important active methanogens in these codigesters despite their low DNA abundance. The lack of aceticlastic *Methanosaeta* in the rRNA libraries of thermophilic digesters and codigesters and the corresponding activity of hydrogenotrophic *Methanoculleus* and *Methanothermobacter* provide further evidence to suggest that SAO is the dominant acetate utilization pathway in those systems. However, further work is needed using isotopically labelled substrates (Schnürer *et al*., 1999) to confirm the dominant methanogenic pathway across the full‐scale digesters.

### Association of microbial community structure and activity with nutrients and biokinetics in full‐scale digesters

**Figure 4:**
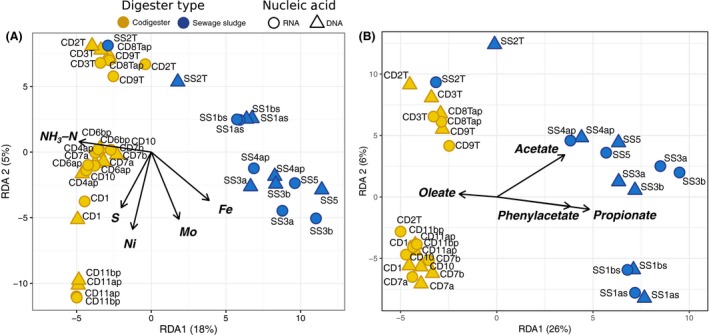
Redundancy analysis (RDA) of OTU read counts in the total microbial community (*Bacteria* + *Archaea*) rRNA gene (DNA) and rRNA (RNA) libraries after variance stabilizing transformation with DESeq2 (Love *et al*., 2014), constrained by significant environmental variables from (A) elemental molar concentrations, and (B) maximum methane production rates (*R*_m_) in batch assays with different substrates. Values in parentheses represent the proportion of total variance explained by each RDA axis. The RDA models were significant at the *P *<* *0.001 level, and the explanatory variables were significant at the *P *<* *0.05 level.

We investigated whether differences in observed biokinetics and nutrient levels were linked to the variation in the abundance (rRNA genes) and activity (rRNA) profiles of microbial populations in the full‐scale digesters. Redundancy analysis (RDA) and permutational model selection showed that five elements—free ammonia (NH_3_‐N), Ni, S, Mo and Fe—could significantly (*P < *0.05) explain the variance in the microbial rRNA gene and rRNA profiles (Fig. 4A). The codigester rRNA and rRNA gene profiles generally associated more closely with higher NH_3_‐N concentrations than the SS digesters based on RDA (Fig. 4A), which is consistent with the higher measured NH_3_‐N in these systems (Table 1). Free ammonia nitrogen is a well‐known growth inhibitor of certain microbial populations in AD, especially aceticlastic methanogens (Schnürer and Nordberg, 2008), and has previously been associated with rRNA gene profiles of anaerobic digesters (De Vrieze *et al*., 2015). Notably, the rRNA profile of the thermophilic sludge digester, SS2T, was more closely associated with higher NH_3_‐N concentrations than its corresponding rRNA gene profile. This discrepancy could be caused by the ‘seeding’ of background microbial DNA through influent sewage sludge, whereas the active community in SS2T was shaped by the selective pressure induced by high temperature and free ammonia (higher free ammonia with higher temperature). S levels were associated with the RNA and DNA profiles of the CD11ap and CD11 bp codigesters, which received slaughterhouse waste that could have contributed to high S levels through protein hydrolysis. Ni and Mo were also associated with the RNA and DNA profiles of the CD11ap and CD11 bp codigesters, which received a proprietary blend of trace elements. Ni and Mo have previously been shown to impact rRNA gene fingerprints of *Bacteria* and *Archaea* communities in digester systems (Feng *et al*., 2010) and are both known to have stimulating effects on methanogenic (Schönheit *et al*., 1979) and syntrophic activities (Plugge *et al*., 2009; Worm *et al*., 2011a,b). Ni is a requirement for factor *F*_430_ involved in methanogenesis (Diekert *et al*., 1981), and Mo is a cofactor of formate dehydrogenase enzymes (Hille, 2002) that enable energy conservation in syntrophs and methanogens (Stams and Plugge, 2009). Ni and Fe are both critical for hydrogenase enzymes used by methanogens (Thauer *et al*., 2010) and syntrophs (Li *et al*., 2011; Worm *et al*., 2011a,b). In this case, Fe was more associated with the RNA and DNA profiles of SS digesters rather than codigesters (Fig. 4A). Fe was previously shown to influence the rRNA gene profiles of digester microbial communities by modulating the Fe:S ratio, which can reduce the concentration of toxic H_2_S (Shakeri Yekta *et al*., 2017). Our findings indicate that the differences in elemental composition among the full‐scale biogas digesters were not only associated with variations in the microbial community rRNA gene abundance, but were also significantly associated with microbial activity based on rRNA gene expression profiles. These results offer additional evidence to support the notion that the activities of digester communities may be adjusted through trace element supplementation (Feng *et al*., 2010; Banks *et al*., 2012; Karlsson *et al*., 2012).

We also investigated whether measured maximum rates of methane production (*R*_m_) from acetate, propionate, phenyl acetate and oleate by the digester contents were associated with microbial population abundance and activity. The *R*_m_ from all substrates could significantly (*P *<* *0.05) explain the variance in the total microbial rRNA gene and rRNA profiles (Fig. 4B). The higher propionate oxidation rates observed in the SS digesters (Fig. 1B) may have been attributed to the higher activity of syntrophic propionate‐oxidizing *Smithella* in those systems compared to the codigesters (Fig. S3). The higher oleic acid conversion rates of the codigesters (Fig. 1B) corresponded with higher rRNA gene and rRNA fractions of LCFA‐degrading *Syntrophomonas* (Fig. S3). Higher concentrations of *Syntrophomonas* have previously been correlated with higher degradation rates of LCFA in anaerobic digestion (Ziels *et al*., 2015, 2016, 2017). Recently, 16S rRNA gene expression of *Syntrophomonas* was correlated with methane production from LCFA‐rich waste (Amha *et al*., 2017). The presence and activity of SAO bacteria in the codigesters were associated with lower maximum rates of acetate consumption (Fig. 1B), which supports previous findings that the biokinetics of SAO may be slower than aceticlastic methanogenesis (Schnürer *et al*., 1999). Overall, the apparent grouping of the full‐scale digester rRNA and rRNA gene profiles based on digester type (Fig. 2) corresponded with different biokinetics of the digester contents, indicating an important relationship between microbial activity levels and digester function. Thus, while AD‐communities may be considered functionally redundant based on substrate type and temperature (Sundberg *et al*., 2013; De Vrieze *et al*., 2015), this redundancy does not necessarily implicate equivalent physiologies in terms of the digester biokinetic capacities (i.e. the different community types had different biokinetics).

### Bacteria and Archaea diversity in full‐scale anaerobic digesters

We assessed whether the levels of diversity in the *Bacteria* and *Archaea* rRNA and rRNA gene libraries were different between the codigesters and municipal SS digesters. Diversity was measured with OTU read counts based on Hill numbers (^*q*^ *D*) at various levels (*q *=* *0, 1, 2), which provides information on both the richness (*q *=* *0) and evenness (*q *>* *0) of the community (Jost, 2006). All levels of Hill numbers were statistically lower (*P *<* *0.05, Welch's *t*‐test) for both *Bacteria* and *Archaea* rRNA and rRNA genes in the codigesters relative to the SS digesters (Fig. S4). Lower Hill numbers with codigestion have been observed in prior works (Ziels *et al*., 2017) and may be caused by the enrichment of specialized microorganisms on influent substrates with certain organic matter compositions. Higher diversity in the SS digesters could also have been attributed to immigration of populations from the PS+WAS feed source, as was shown to occur in other biological wastewater treatment processes (Wells *et al*., 2014). There were no significant differences within intragroup diversity (i.e. within codigesters and municipal sludge digesters *per se*) for the rRNA and rRNA gene libraries at all Hill numbers (*q *=* *0, 1, 2; *P *>* *0.1, *t*‐test), indicating that the diversity in DNA libraries generally reflected the diversity of the active communities (RNA). An exception to this general trend was the thermophilic SS2T digester, which had lower diversity in its rRNA library relative to its rRNA gene library at all levels of Hill numbers libraries for both *Bacteria* and *Archaea* (Fig. S4). Similarly, the thermophilic codigesters, CD2T, CD3T and CD9T, had lower *Bacteria* rRNA diversity relative to rRNA genes at all Hill numbers. Thermophilic digesters (both in SS and CD) had significantly lower diversity at all Hill numbers in rRNA profiles than mesophilic digesters (*P* < 0.005, Welch's *t*‐test). This lower rRNA diversity under thermophilic conditions could be caused by a greater selective growth pressure leading to a community with fewer active members, as was suggested in prior works based on rRNA genes (Levén *et al*., 2007; Pervin *et al*., 2013). Further research is thus warranted to monitor the ratio of diversity in rRNA to rRNA genes during periods of process instability to determine its utility as a potential monitoring indicator for microbial stress in AD.

### Co‐occurrence of microbial rRNA signatures in full‐scale digesters

**Figure 5:**
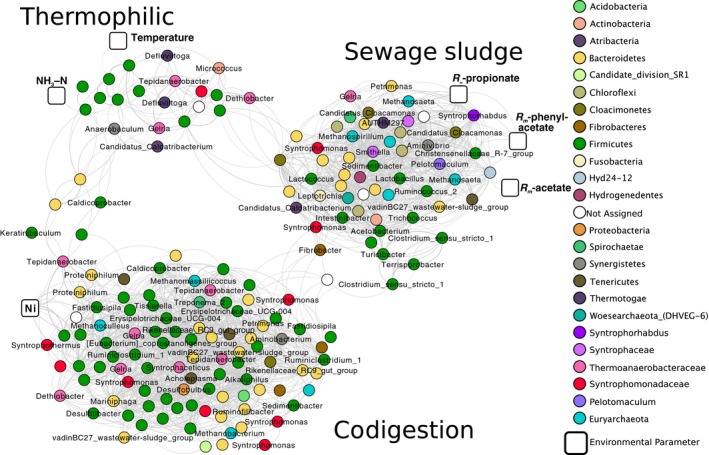
Co‐occurrence network based on correlation of OTU read counts in 16S rRNA gene expression profiles across the 20 full‐scale anaerobic digesters. Nodes are coloured by taxonomy with labelled genera names when possible, and edges are shown as grey lines. The thickness of the edge is proportional to the *r* value of the correlation (from 0.8 to 0.99). Syntrophic bacteria groups and archaeal groups are depicted in pinks/reds and turquoise respectively. Boxed nodes represent environmental parameters.

A total of 2339 statistically significant (*P *<* *0.001) pairs (i.e. positive edges) were detected among 174 OTUs (i.e. nodes) based on correlation analysis of their 16S rRNA gene expression profiles (Fig. 5). Three main subclusters were detected by dissecting the overall co‐occurrence network (Table S4). Inspecting the phylogenetic distribution of the taxa within each subcluster revealed that their arrangement corresponded to the major groupings observed by PCA (Fig. 2) reflecting the three main digester types (i.e. mesophilic codigesters, mesophilic SS digesters and thermophilic digesters). This was further supported by the inclusion of nodes for digester temperature and NH_3_‐N within one subcluster, a node for Ni within a second subcluster and a node for *R*_m_‐propionate, *R*_m_‐phenylacetate and *R*_m_‐acetate in the third subcluster (Fig. 5).

The subcluster associated with *R*_m_‐propionate, *R*_m_‐phenyl acetate and *R*_m_‐acetate contained OTUs belonging to *Methanosaeta, Methanospirillum, Smithella, Syntrophaceae, Candidatus* Cloacamonas, *Pelotomaculum* and *Syntrophorhabdus*, which were representative of rRNA profiles in the SS digesters (Fig. S3). Notably, the co‐occurrence of potential propionate‐oxidizing OTUs belonging to *Pelotomaculum, Candidatus* Cloacimonas and *Smithella* (Liu *et al*., 1999; Imachi *et al*., 2002) and aromatics‐degrading *Syntrophorhabdus* (Qiu *et al*., 2008) and *Syntrophaceae* could explain the higher propionate and phenyl acetate degradation kinetics observed with SS digester contents (Fig. 1B). The ability of *Candidatus* Cloacimonas acidaminovorans to syntrophically degrade propionate was hypothesized based on in‐silico genome analysis (Pelletier *et al*., 2008). The first neighbours of the *R*_m_‐propionate node included a *Methanosaeta‐*OTU, a *Candidatus* Cloacimonas‐OTU, a *Syntrophorhabdus*‐OTU and a *Syntrophaceae‐*OTU, suggesting their potential involvement in syntrophic propionate degradation (Schink, 1997). OTUs assigned to the uncultured archaeal groups of *Woesearchaeota* and the Terrestrial Miscellaneous Euryarchaeotic Group (TMEG) also shared edges with the *R*_m_‐propionate node. It was inferred that members of *Woesearchaeota* may have fermentative and symbiotic lifestyles based on genome reconstruction from metagenomes (Castelle *et al*., 2015), but their role in anaerobic digesters is unknown and warrants further investigations based on these findings that their activity correlates with higher substrate turnover rates.

The second subcluster was associated with Ni levels and contained OTUs within *Methanoculleus, Syntrophomonadaceae, Tepidanaerobacter, Syntrophaceticus* and *Gelria,* which all had significantly higher rRNA gene expression levels in the municipal codigesters in comparison with SS digesters (*P *<* *0.05, Welch's *t*‐test; Fig. S3). The first neighbours of the Ni node included several potential SAO OTUs, including *Thermoanaerobacteraceae* and *Tepidanaerobacter*. SAO activity relies on a Ni‐containing CO‐dehydrogenase (Müller *et al*., 2013), and Ni addition was previously shown to enhance acetate degradation in SAO‐dominated systems (Karlsson *et al*., 2012). These results suggest that the activity of SAO is positively associated with Ni levels, but the mechanism for this association needs further confirmation by enzymatic and transcriptomic‐based research.

The third cluster was associated with higher NH_3_‐N and temperature, which were characteristic of the thermophilic digesters (Table 1). The OTU sharing an edge with the NH_3_‐N node belonged to the uncultured group of *Firmicutes* OPB54. In a recent study, *Firmicutes* OPB54‐assigned OTUs increased in abundance concurrently with a shift towards SAO in high‐ammonia digesters (Müller *et al*., 2016). Further directed studies are needed to examine the ecophysiology of *Firmicutes* OPB54 in high ammonia digesters, as it was previously suggested that SAO can be conducted by diverse uncultured bacterial lineages of which little genetic information is known (Werner *et al*., 2014). The first neighbours of the temperature node included OTUs assigned to *Defluviitoga* and *Tepidanaerobacter* (Fig. 5), which agrees with the higher rRNA gene expression of those genera in the thermophilic digesters (Fig. S3). The thermophilic subcluster had the lowest edge count of all subclusters (Table S4). Similarly, edge counts were negatively correlated to temperature in co‐occurrence networks based on rRNA profiles in anaerobic digesters (Lin *et al*., 2017), which could potentially be explained by a lower diversity at higher temperatures (Fig. S4) limiting the number of species interactions. However, richness and evenness were not correlated with positive edge percentages in networks across various environments (Faust *et al*., 2015), and thus, the lower edge count in the thermophilic subcluster could be attributed to other community interactions.

Notably, syntrophic and methanogenic taxa were prevalent throughout the overall network, indicating that such obligate energy‐sharing partnerships play critical roles in stabilizing the digester microbiome. An exception was the lack of a methanogenic taxon in the thermophilic subcluster. Metabolic interdependencies, such as syntrophy, were identified as a major driver of species co‐occurrence across a diverse set of microbial ecosystems (Zelezniak *et al*., 2015). The high representation of syntrophs and methanogens in the co‐occurrence network may also be attributed to their metabolic specialization (Sieber *et al*., 2012), suggesting that their ecological niches are not easily filled by other taxa (Werner *et al*., 2011). Yet, recent work has shown high strain variability within digester syntrophic communities under different feeding strategies (Ziels *et al*., 2017, 2018), indicating that selective forces may still govern the syntrophic community structure in different digester environments.

Overall, co‐occurrence analysis of 16S rRNA gene expression profiles revealed three distinct subclusters of active microbes within different digester types, which were linked to differences in their biokinetics and elemental compositions. While these results are indicative of alternative stability states or high functional redundancy (Shade *et al*., 2012) for digester microbiomes based on different environmental conditions, the co‐occurrence data alone are purely observational and do not provide a mechanistic understanding of the types of interactions between species. However, these findings warrant further longitudinal studies with planned perturbations and/or physiological investigations using metagenomics and metatranscriptomics to elucidate the nature of interactions among microbes based on varying digester environments. For instance, such experiments should be focused to elucidate the role of uncultured archaeal groups in SS digesters, as well as to illuminate the nature of interactions between micro‐ and macronutrients and uncultivated SAO taxa to develop solutions for improved digester capacity and methane production efficiency.

## Experimental procedures

### Sample sources and analysis of nutrient compositions

Digestate samples were collected from the effluent of 20 full‐scale anaerobic digesters for parallel biokinetic profiling, elemental analysis and sequencing of 16S rRNA and rRNA genes. The operational parameters of each digester including influent substrate composition, temperature, organic loading rate (OLR) and hydraulic retention time (HRT) are presented in Table 1.

Samples were analysed for elemental contents including NH_4_‐N, Cl, P, Al, B, Ca, Co, Cu, Fe, K, Mg, Mn, Mo, Na, Ni, Se, S, Ti, V, W and Zn by Eurofin Environment Testing Sweden AB (Sweden). Elemental analyses were carried out per the Swedish Standard Method SS028150‐2, except for NH_4_‐N and Cl, which were determined per the Standard Methods 4500‐ammonia and 4500‐chloride. The measured elemental concentrations and analytical uncertainties are presented in the (Table S3). Free ammonia levels were calculated based on measured levels of NH_4_‐N, pH and temperature for each digester.

### Batch assays for substrate conversion biokinetics

Degradation biokinetics of acetate, propionate, phenyl acetate and oleic acid by the digester contents were analysed in batch assays according to Karlsson *et al*. (2012). In short, 15 ml of digestate samples, corresponding to 0.12–0.68 g VS, was inoculated in 330 ml gas‐tight bottles containing 135 ml of growth medium at 37°C. A detailed description of composition of the growth medium is found in Karlsson *et al*. (1999). The headspace was purged with a gas mixture of 80% nitrogen and 20% carbon dioxide. Acetate, propionate, phenyl acetate and oleic acid were separately added to triplicate bottles with final concentrations of 15, 15, 5 and 5 mM respectively. Triplicate control assays with only digestate were used to determine the methane production from the background organic matter of the samples. Volumetric biogas production was determined by measuring the gas pressure in the bottles using a digital pressure meter (Testo 3123; Testo, Sparta, NJ, USA) at 37°C. Gas samples (1 ml) were also collected from the bottles at nine occasions during 240 h incubation for methane concentration measurements using GC‐FID (HP 5880A; Hewlett Packard, Palo Alto, California, USA). Total solid and VS contents of the samples were determined per the Swedish Standard Method (SS‐028113).

The maximum methane production rates (*R*_m_) (ml CH_4_ g VS^−1^ h^−1^) in batch assays were calculated by fitting a Gompertz model to the accumulated methane production (*M*) curve versus incubation time (*t*):

$$M\left(t\right)=P\cdot \text{exp}[-\exp\left[\frac{R_{m}\cdot e}{P}\right]\left(\lambda -t\right)+1]]$$

Parameters *P* and λ are model parameters termed as methane production potential (ml CH_4_) and lag phase (*h*) respectively. Methane volumes refer to standard temperature and pressure. Model fitting was conducted in R version 3.3. Fitted *R*_m_ values are presented in Table S2, and example profiles of the model fitting are shown in Fig. S5.

### DNA and RNA extraction, 16S rRNA amplicon sequencing and statistics

A detailed protocol for sample processing for DNA and RNA extraction, reverse transcription to complimentary DNA (cDNA) and PCR amplification is provided in the Supplemental Methods (found in Supporting Information). Briefly, PCR amplification of DNA and cDNA was conducted according to Sundberg *et al*. (2013) with primers 515F and a modified 915R (Casamayor *et al*., 2002). The sequence library was prepared according to Ziels *et al*. (2015). The amplicon libraries were sequenced on an Illumina MiSeq in 2 × 250 bp mode at the University of Copenhagen Molecular Microbial Ecology Lab. Raw sequence reads have been submitted to the NCBI SRA database under BioProject PRJNA402061.

16S rRNA and rRNA gene reads were quality filtered and trimmed and denoised into exact sequences using DADA2 (Callahan *et al*., 2016). Forward and reverse reads were initially truncated at 240 bp and then trimmed by the first 28 and 35 bp, respectively, to remove primers and adapters. A maximum expected error of 2 was used to remove low‐quality reads. After denoising and merging the forward and reverse reads, sequences were clustered into OTUs using UPARSE (Edgar, 2013) based on a 99% similarity threshold, resulting in 4725 OTUs. Representative sequences of each OTU were taxonomically classified against the SILVA SSU Ref NR dataset (v.123) using the RDP classifier (Wang *et al*., 2007). Denoising and error‐correcting sequences with DADA2 before OTU clustering allowed for a smaller clustering radius to be used without generating spurious or erroneous OTUs (Callahan *et al*., 2016), thus, enabling a fine‐scale analysis of community structure. A detailed description of read processing and statistical analysis is given in the Supplementary Information.

OTU relative abundance values were calculated with DESeq2 (Love *et al*., 2014) normalized sequence counts, which account for the different sequencing depths among samples. Diversity was presented using Hill diversity numbers (^*q*^ *D*) of orders *q* = 0, 1 and 2 (Jost, 2006) using OTU read counts. Diversity of order 0 (i.e. ^*0*^ *D*) is equal to species richness, ^1^ *D* is equal to exp(Shannon entropy), and ^2^ *D* is equivalent to 1/(Simpson concentration) (Jost, 2006).

Principal components analysis (PCA) was conducted using OTU read counts after variance stabilizing (VS) transformation with DESeq2 (Love *et al*., 2014). Redundancy analysis (RDA) was used to summarize linear relationships between OTU read counts (VS‐transformed) and maximum methane production rates (*R*_m_) and elemental concentrations in the digesters. For the elemental dataset, multicollinearity was identified using Pearson correlation, and variables with *r* values above 0.7 were removed prior to RDA. Model selection was conducted using forward–backward selection with the *ordistep* function within the vegan package v.2.4.2 (Oksanen *et al*., 2007) for R version 3.3. Significance of the RDA solution and the selected exploratory variables were assessed through permutational ANOVA in vegan. Differences in biokinetic and nutrient profiles between the digester types were assessed using analysis of similarities (ANOSIM) based on Bray–Curtis distances with the *anosim* function within the vegan package in R.

A co‐occurrence matrix was constructed by calculating correlations between OTU rRNA read counts based on an ensemble approach of Spearman, Pearson and Kendall correlations using CoNet v.1.1.1 (Faust and Raes, 2016). A statistically significant correlation was defined by an *r* value >0.75 and *P *<* *0.01 after multiple‐test correction with the Bonferroni procedure (Faust and Raes, 2016). Only OTUs that had >5 read counts in seven digester samples were included in the correlation analysis to reduce spurious correlations (Berry and Widder, 2014), and only positive associations were included in the subsequent network. Correlations between OTUs and environmental parameters were also considered in the network analysis, including NH_3_‐N, Fe, Ni, Mo, temperature and *R*_m_ values of acetate, propionate, oleate and phenyl acetate. Only NH_3_‐N, Ni, temperature, *R*_m_‐phenyl acetate, *R*_m_‐acetate and *R*_m_‐propionate had significant correlations (*r *>* *0.75, *P *<* *0.01). The network was visualized and analysed for topological features in Cytoscape v. 3.5.1 (Shannon *et al*., 2003). Distinct clusters within the network were detected using the OH‐PIN algorithm within CytoCluster.

## Conflict of interest

The authors declare no conflict of interests.

## Supporting information


**Fig. S1.** (A) NMDS plot based on Bray‐Curtis distances of the element concentrations in the 20 digesters.
**Fig. S2.** Relative sequence fractions of: (A) the 12 most abundant *Bacteria* phyla in the 16S rRNA and rRNA gene profiles of all full‐scale digesters; (B) the 4 most abundant *Archaea* phyla in the 16S rRNA and rRNA gene profiles of all full‐scale digesters.
**Fig. S3.** Relative sequence fractions of: (A) the 25 most abundant *Bacteria* genera in the 16S rRNA and rRNA gene profiles of all full‐scale digesters; (B) the 15 most abundant *Archaea* genera in the 16S rRNA and rRNA gene profiles of all full‐scale digesters.
**Fig. S4.** Hill diversity numbers (^*q*^ *D*) in 16S rRNA and rDNA profiles for *Bacteria* at orders of (A) *q *= 0.
**Fig. S5.** Example plots of fitting the Gompertz model (dashed lines) to cumulative methane production in the batch kinetics assays inoculated with sludge samples from SS1bs (A) and CD1 (B) reactors.
**Table S1.** Volatile fatty acid profiles of sludge sampled from full‐scale digesters.
**Table S2.** Values of the maximum methane production rate (*R*m) based on fitting a Gompertz model to cumulative methane production over time in batch assays with the various digester sludges spiked with acetate, propionate, oleate, and phenyl acetate, as well as un‐amended controls.
**Table S3.** Elemental concentrations (M) and uncertainties for the 20 full‐scale anaerobic digester biomass samples.
**Table S4.** Network topology parameters estimated in Cytoscape v. 3.5.1.
