# Supplementary material for: Microbial rRNA gene expression and co‐occurrence profiles associate with biokinetics and elemental composition in full‐scale anaerobic digesters
Source: Microb Biotechnol. 2018 Apr 6;11(4):694–709. doi: 10.1111/1751-7915.13264 (PMC6011980; doi:10.1111/1751-7915.13264)
Supplement: Supplementary file 1 — Fig. S1. (A) NMDS plot based on Bray‐Curtis distances of the element concentrations in the 20 digesters. Fig. S2. Relative sequence fractions of: (A) the 12 most abundant Bacteria phyla in the 16S rRNA and rRNA gene profiles of all full‐scale digesters; (B) the 4 most abundant Archaea phyla in the 16S rRNA and rRNA gene profiles of all full‐scale digesters. Fig. S3. Relative sequence fractions of: (A) the 25 most abundant Bacteria genera in the 16S rRNA and rRNA gene profiles of all full‐scale digesters; (B) the 15 most abundant Archaea genera in the 16S rRNA and rRNA gene profiles of all full‐scale digesters. Fig. S4. Hill diversity numbers (q D) in 16S rRNA and rDNA profiles for Bacteria at orders of (A) q = 0. Fig. S5. Example plots of fitting the Gompertz model (dashed lines) to cumulative methane production in the batch kinetics assays inoculated with sludge samples from SS1bs (A) and CD1 (B) reactors. Table S1. Volatile fatty acid profiles of sludge sampled from full‐scale digesters. Table S2. Values of the maximum methane production rate (Rm) based on fitting a Gompertz model to cumulative methane production over time in batch assays with the various digester sludges spiked with acetate, propionate, oleate, and phenyl acetate, as well as un‐amended controls. Table S3. Elemental concentrations (M) and uncertainties for the 20 full‐scale anaerobic digester biomass samples. Table S4. Network topology parameters estimated in Cytoscape v. 3.5.1. [file MBT2-11-694-s001.pdf]

Supplemental Information for:

Microbial rRNA gene expression and co-occurrence profiles associate with biokinetics and elemental composition in full-scale anaerobic digesters

Ryan M. Ziels[a,*], Bo H. Svensson[b,c], Carina Sundberg[b], Madeleine Larsson[b,c], Anna Karlsson[d], Sepehr Shakeri Yekta[b,c]

[a] Department of Civil Engineering, The University of British Columbia, Vancouver, Canada

[b] Department of Thematic Studies-Environmental Change, Linköping University, Linköping, Sweden

[c] Biogas Research Center, Linköping University, Linköping, Sweden

[d] Scandinavian Biogas Fuels AB, Stockholm, Sweden

Number of Pages: 12

Number of Tables: 4

Number of Figures: 5

Supplemental Methods: DNA and RNA extraction, 16S rRNA amplicon sequencing and statistics

15 mL aliquots of digester sludge were immediately transferred into sterilized tubes and frozen on dry ice during transport from the full-scale plants, and were transferred into storage at -80 °C within 24 hours of collection. For DNA and RNA extractions, the samples were briefly thawed at room temperature, and were shaken before being transferred to extraction tubes. DNA was extracted in duplicate from 0.4 g of digester biomass using the FastDNA® SPIN Kit for Soil (MP Biomedicals, LLC; Solon, Ohio) per the manufacturer's instructions. RNA was extracted in duplicate from 0.8 g digester biomass by bead-beating with the FastRNA® Pro Soil-Direct Kit (MP Biomedicals), centrifuging for 40 s at 6000 x *g*, and thereafter 300 µl supernatant was treated per the manufacturer's instructions. Quantification of DNA and RNA was carried out according to Moestedt *et al.* (2016). Thereafter the duplicate DNA and RNA extractions were separately pooled together at equal volumes, resulting in a single DNA and RNA sample for each digester. Residual DNA in the RNA samples was removed by Ambion®TURBO DNA-*free*™ Kit (Invitrogen, Lidingö, Sweden). DNA-free RNA was reverse transcribed (RT) to complementary DNA (cDNA) using the Illustra™ Ready-to-Go RT-PCR Beads (GE-Healthcare, Uppsala, Sweden). cDNA and DNA samples were stored at -20°C.

Duplicate PCR amplifications (extracted DNA concentrations were between 64-110 µg mL^{-1} and cDNA concentrations were between 5-29 µg mL^{-1}) were conducted as described in Sundberg et al., (2013) except for the reverse primer used, 915R (GTGCTCCCCCGCCAATTCCT) (Casamayor et al., 2002). The duplicate PCR products were pooled before purification by the High Pure PCR Cleanup Micro Kit per the manufacturer's instructions (Roche Diagnostics GmbH, Mannheim, Germany) and quantified as described in Sundberg et al., (2013). Library preparation was conducted according to Ziels et al. (2015). The amplicon libraries were sequenced on an

Illumina MiSeq in 2x250 bp mode at the University of Copenhagen Molecular Microbial Ecology Lab.

The DADA2 pipeline for denoising 16S rRNA amplicons and taxonomic classification was conducted as described in Supplementary File 2.

Supplemental Table S1. Volatile fatty acid profiles of sludge sampled from full-scale digesters.

Digester	Acetic acid (mM)	Propionic acid (mM)	Iso-butyric acid (mM)	Butyric acid (mM)	Iso-valeric acid (mM)	Valeric acid (mM)	Iso-caproic acid (mM)	Caproic acid (mM)	Heptanoic acid (mM)
CD1	0.48	0.12	0.00	0.00	0.00	0.00	0.00	0.00	0.00
CD2T	2.95	0.37	0.03	0.01	0.03	0.00	0.00	0.00	0.00
SS1as	0.17	0.02	0.00	0.00	0.00	0.00	0.00	0.00	0.00
SS1bs	0.09	0.01	0.00	0.00	0.00	0.00	0.00	0.00	0.00
SS2T	0.15	0.01	0.00	0.00	0.00	0.00	0.00	0.00	0.00
CD3T	1.25	7.97	0.52	0.01	0.13	0.01	0.05	0.00	0.00
CD4ap	7.17	1.19	0.12	0.05	0.15	0.01	0.01	0.00	0.00
SS3a	0.29	0.06	0.01	0.00	0.01	0.00	0.00	0.00	0.00
SS3b	0.13	0.03	0.00	0.00	0.00	0.00	0.00	0.00	0.00
CD6ap	12.52	3.11	0.50	0.33	0.70	0.16	0.05	0.01	0.00
CD6bp	13.55	3.40	0.50	0.36	0.76	0.17	0.06	0.02	0.01
CD5ap	2.33	0.93	0.04	0.02	0.04	0.01	0.01	0.00	0.00
CD5bp	2.47	0.87	0.04	0.01	0.02	0.01	0.00	0.00	0.00
SS4ap	0.12	0.02	0.00	0.00	0.01	0.00	0.00	0.00	0.00
SS4bp	0.10	0.02	0.00	0.00	0.00	0.00	0.00	0.00	0.00
CD7a	3.28	1.85	0.11	0.01	0.39	0.01	0.01	0.00	0.00
CD7b	5.41	3.25	0.60	0.08	0.87	0.08	0.04	0.00	0.00
SS5	0.36	0.18	0.03	0.01	0.06	0.01	0.00	0.00	0.00
CD8Tap	0.42	0.05	0.01	0.00	0.02	0.00	0.00	0.00	0.00
CD8Tbp	2.12	0.07	0.01	0.01	0.01	0.00	0.00	0.00	0.00
CD9T	2.30	3.10	0.47	0.03	0.14	0.03	0.02	0.00	0.00
CD10	3.46	0.34	0.05	0.01	0.06	0.00	0.00	0.00	0.00
CD11ap	0.75	0.07	0.01	0.00	0.01	0.00	0.00	0.00	0.00
CD11bp	0.10	0.01	0.00	0.10	0.00	0.00	0.00	0.00	0.00

Supplemental Table S2. Values of the maximum methane production rate (R_m) based on fitting a Gompertz model to cumulative methane production over time in batch assays with the various digester sludges spiked with acetate, propionate, oleate, and phenyl acetate, as well as un-amended controls. Values in parentheses represent the standard error based on the model fit in R v.3.3.0.

Digester	R_m Acetate ($mL\ CH_4\ g\ VS^{-1}\ hr^{-1}$)	R_m Propionate ($mL\ CH_4\ g\ VS^{-1}\ hr^{-1}$)	R_m Oleate ($mL\ CH_4\ g\ VS^{-1}\ hr^{-1}$)	R_m Phenyl Acetate ($mL\ CH_4\ g\ VS^{-1}\ hr^{-1}$)	R_m Control ($mL\ CH_4\ g\ VS^{-1}\ hr^{-1}$)
CD1	0.6 (0.04)	0.8 (0.09)	0.4 (0.02)	0.3 (0.03)	0.3 (0.02)
CD2T	2.7 (0.20)	1.2 (0.06)	1.3 (0.11)	0.8 (0.11)	1.3 (0.10)
CD3T	1.9 (0.14)	1.0 (0.06)	0.3 (0.05)	0.9 (0.08)	1.0 (0.09)
CD4ap	0.7 (0.05)	0.9 (0.03)	0.9 (0.04)	0.6 (0.05)	0.5 (0.04)
CD5ap	0.9 (0.04)	0.8 (0.05)	0.9 (0.04)	0.6 (0.03)	0.5 (0.03)
CD5bp	0.9 (0.03)	0.7 (0.08)	0.7 (0.03)	0.6 (0.02)	0.5 (0.02)
CD7a	0.6 (0.05)	0.4 (0.03)	0.3 (0.04)	0.3 (0.03)	0.4 (0.04)
CD7b	0.7 (0.09)	0.7 (0.07)	0.6 (0.09)	0.5 (0.07)	0.6 (0.09)
CD8Tap	1.7 (0.13)	1.1 (0.04)	0.9 (0.11)	0.5 (0.08)	0.6 (0.07)
CD9T	1.2 (0.14)	0.8 (0.09)	0.9 (0.23)	0.5 (0.13)	0.4 (0.06)
CD10	0.8 (0.03)	1.2 (0.03)	0.4 (0.05)	0.5 (0.03)	0.5 (0.03)
CD11ap	0.9 (0.05)	0.6 (0.03)	0.1 (0.02)	0.6 (0.04)	0.5 (0.03)
CD11bp	0.9 (0.06)	0.7 (0.07)	0.1 (0.02)	0.7 (0.05)	0.4 (0.05)
SS1as	2.8 (0.21)	3.7 (0.16)	0.8 (0.08)	1.9 (0.22)	1.8 (0.19)
SS1bs	3.2 (0.17)	3.3 (0.12)	1.1 (0.24)	2.1 (0.15)	1.6 (0.09)
SS2T	3.5 (0.39)	2.1 (0.17)	1.1 (0.19)	1.6 (0.22)	1.6 (0.17)
SS3a	3.5 (0.19)	3.2 (0.28)	2.0 (0.52)	1.8 (0.21)	1.4 (0.21)
SS3b	2.7 (0.22)	2.9 (0.31)	0.3 (0.09)	1.8 (0.14)	1.1 (0.17)
SS4ap	2.5 (0.28)	1.9 (0.08)	0.9 (0.16)	1.4 (0.11)	1.0 (0.18)
SS5	2.5 (0.34)	2.2 (0.21)	0.1 (0.02)	1.3 (0.22)	1.3 (0.29)

Supplemental Table S3. Elemental concentrations (M) and uncertainties for the 20 full-scale anaerobic digester biomass samples.

	NH_4-N	Cl	P	Al	B	Ca	Co	Cu	Fe	K	Mg	Mn	Mo	Na	Ni	Se	S	Ti	V	W	Zn
**Analytical Uncertainty**	10%	8%	15%	20%	15%	15%	20%	15%	15%	20%	15%	15%	20%	20%	15%	20%	20%	15%	15%	25%	15%
SS1as	5.7E-02	1.4E-02	1.7E-02	5.5E-03	8.7E-05	1.2E-02	1.0E-06	9.8E-05	1.8E-02	2.2E-02	3.2E-03	5.9E-05	8.9E-07	5.9E-03	4.3E-06	2.9E-07	8.4E-03	3.3E-05	6.8E-06	2.1E-07	1.1E-04
SS1bs	5.7E-02	1.4E-02	1.5E-02	5.4E-03	8.1E-05	1.2E-02	9.7E-07	9.0E-05	1.6E-02	1.6E-02	2.8E-03	5.5E-05	8.5E-07	5.9E-03	3.6E-06	3.1E-07	6.3E-03	3.5E-05	6.0E-06	2.1E-07	1.0E-04
SS3a	7.1E-02	1.4E-02	1.9E-02	1.5E-02	5.5E-05	2.3E-02	1.4E-06	1.8E-04	1.2E-02	4.8E-03	5.2E-03	7.3E-05	2.6E-06	7.5E-03	7.2E-06	1.3E-06	1.5E-02	8.3E-05	5.9E-06	3.4E-07	3.1E-04
SS3b	9.2E-02	1.4E-02	4.0E-02	1.5E-02	6.6E-05	2.1E-02	1.5E-06	2.1E-04	3.3E-02	8.0E-03	7.5E-03	7.6E-05	2.6E-06	6.8E-03	8.2E-06	1.1E-06	1.3E-02	4.0E-04	7.6E-06	5.0E-07	2.7E-04
SS4ap	5.7E-02	1.4E-02	3.6E-02	1.7E-02	7.8E-05	2.2E-02	1.5E-06	2.0E-04	3.9E-02	5.4E-03	5.7E-03	1.8E-04	2.2E-06	5.8E-03	8.3E-06	1.1E-06	1.3E-02	1.4E-04	1.3E-05	7.0E-07	3.5E-04
SS5	7.1E-02	1.4E-02	3.2E-02	9.0E-03	7.1E-05	2.2E-02	2.0E-06	1.6E-04	4.2E-02	3.8E-03	3.8E-03	1.3E-04	2.3E-06	3.8E-03	9.3E-06	8.9E-07	1.0E-02	8.7E-05	8.8E-06	3.6E-07	2.6E-04
SS2T	8.5E-02	1.4E-02	3.5E-02	2.9E-02	4.0E-04	1.6E-02	1.2E-06	1.1E-04	2.7E-02	6.0E-03	5.5E-03	1.4E-04	1.4E-06	8.1E-03	5.9E-06	6.2E-07	9.6E-03	4.2E-05	6.8E-06	5.4E-07	2.0E-04
CD2T	1.4E-01	3.4E-02	7.8E-03	3.5E-03	1.1E-04	1.1E-02	1.0E-06	1.4E-05	2.7E-03	1.9E-02	3.1E-03	5.7E-05	2.1E-06	2.9E-02	1.8E-06	3.0E-07	3.6E-03	6.0E-06	2.2E-06	7.2E-08	6.2E-05
CD3T	2.6E-01	6.1E-02	2.6E-02	5.3E-03	1.1E-04	2.2E-02	1.8E-06	6.6E-05	9.3E-03	7.2E-02	7.4E-03	1.9E-04	1.7E-06	4.1E-02	3.3E-06	1.1E-06	1.4E-02	1.1E-05	8.2E-06	2.5E-07	2.1E-04
CD8Tap	8.5E-02	3.4E-02	1.1E-02	2.6E-03	9.4E-05	2.4E-02	1.7E-06	3.9E-05	1.0E-03	3.1E-02	5.4E-03	9.5E-05	8.1E-07	4.2E-02	4.3E-06	3.2E-07	4.4E-03	9.2E-06	4.9E-06	1.6E-07	9.5E-05
CD9T	1.5E-01	2.8E-02	7.6E-03	1.0E-03	5.2E-05	1.1E-02	6.9E-07	1.1E-05	6.4E-04	2.4E-02	3.4E-03	3.7E-05	5.3E-07	3.0E-02	1.7E-06	2.1E-07	3.0E-03	1.1E-05	3.2E-06	2.3E-07	4.9E-05
CD1	3.5E-01	1.7E-01	4.6E-02	1.6E-04	1.3E-04	5.8E-03	8.0E-06	2.4E-05	4.6E-02	9.9E-02	5.7E-03	1.3E-04	2.1E-06	2.8E-02	6.1E-06	7.4E-07	6.4E-02	7.6E-06	1.3E-05	3.6E-07	2.5E-04
CD4ap	2.1E-01	4.8E-02	1.8E-02	3.0E-03	7.8E-05	2.1E-02	1.4E-06	7.7E-05	7.4E-03	4.6E-02	4.3E-03	1.5E-04	1.5E-06	4.1E-02	3.3E-06	1.3E-06	1.1E-02	1.5E-05	6.6E-06	2.1E-07	2.6E-04
CD6ap	2.1E-01	4.5E-02	1.5E-02	8.6E-04	1.1E-04	4.1E-02	1.8E-06	1.0E-04	1.0E-02	5.0E-02	1.0E-02	4.8E-04	1.3E-06	4.3E-02	3.2E-06	7.1E-07	1.1E-02	1.7E-05	8.3E-06	2.6E-07	2.6E-04
CD6bp	1.9E-01	4.8E-02	1.6E-02	9.8E-04	1.1E-04	4.6E-02	1.9E-06	1.1E-04	1.1E-02	5.4E-02	1.1E-02	5.2E-04	1.4E-06	4.7E-02	3.3E-06	8.5E-07	1.2E-02	1.8E-05	8.7E-06	2.7E-07	2.7E-04
CD7a	1.8E-01	3.4E-02	2.6E-02	2.2E-02	1.0E-04	2.1E-02	1.9E-06	3.3E-05	8.9E-03	3.6E-02	7.7E-03	1.4E-04	1.3E-06	3.7E-02	3.8E-06	7.1E-07	1.5E-02	5.1E-05	1.4E-05	2.8E-07	1.9E-04
CD7b	1.4E-01	3.3E-02	2.4E-02	1.9E-02	1.1E-04	2.3E-02	2.0E-06	3.8E-05	6.8E-03	3.9E-02	8.3E-03	1.4E-04	1.6E-06	3.2E-02	3.4E-06	7.4E-07	1.4E-02	3.7E-05	1.2E-05	2.9E-07	2.1E-04
CD10	1.8E-01	3.9E-02	1.3E-02	3.0E-03	1.7E-04	3.1E-02	1.8E-06	3.9E-05	1.8E-03	7.5E-02	1.1E-02	1.4E-04	2.0E-06	4.1E-02	5.1E-06	5.4E-07	6.9E-03	3.5E-05	8.4E-06	2.7E-07	1.1E-04
CD11ap	3.3E-01	8.1E-02	3.3E-02	1.3E-03	1.3E-04	1.3E-02	8.2E-06	3.8E-05	2.3E-02	3.9E-02	3.2E-03	1.6E-04	2.4E-06	1.0E-01	3.7E-05	9.9E-07	2.6E-02	2.1E-05	1.1E-05	3.6E-07	2.4E-04
CD11bp	3.2E-01	8.1E-02	3.2E-02	1.3E-03	1.3E-04	1.3E-02	7.7E-06	3.5E-05	2.2E-02	4.0E-02	2.9E-03	1.5E-04	2.5E-06	1.1E-01	3.6E-05	8.9E-07	2.5E-02	2.1E-05	1.1E-05	3.5E-07	2.3E-04

Supplemental Table S4. Network topology parameters estimated in Cytoscape v. 3.5.1.

**Topological Parameter**	**Overall Network**	**Codigester-associated Sub-cluster**	**SS-associated Sub-cluster**	**Thermophilic-associated Sub-cluster**
Node Count	181	96	63	23
Edge Count	2339	1423	817	104
Clustering Coefficient	0.617	0.63	0.736	0.65
Network Centralization	0.198	0.339	0.283	0.366
Network Diameter	7	4	4	3
Network Density	0.121	0.268	0.339	0.348
Average Path Length	3.31	1.94	1.85	1.76

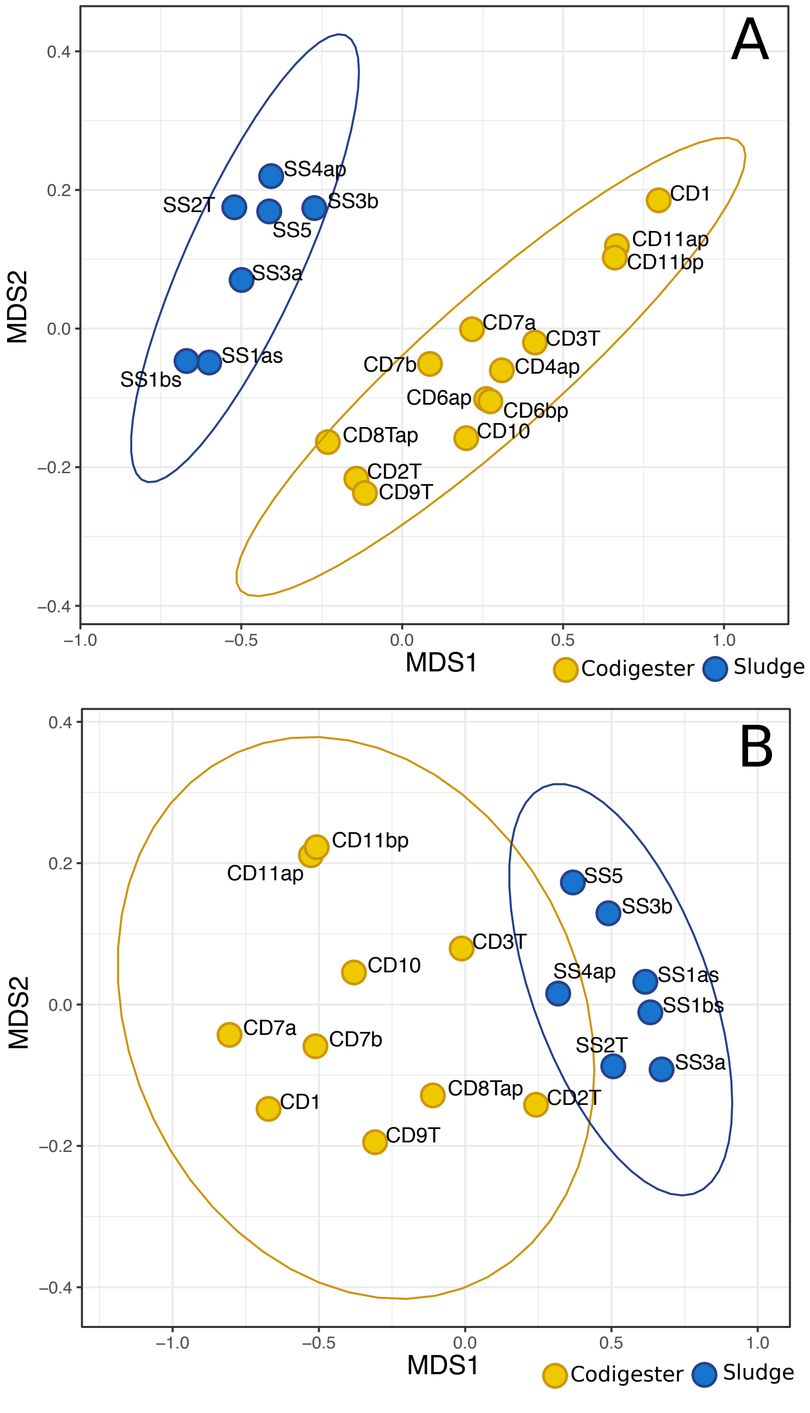

Supplemental Figure S1. (A) NMDS plot based on Bray-Curtis distances of the element concentrations in the 20 digesters. (B) NMDS plot based on Bray-Curtis distances of fitted R_m parameter values for the four substrates for all digester biomasses. The total stress of both NMDS plots were less than 0.02, and ellipses represent 95% confidence intervals around the centroid of each digester group (codigesters and sludge digesters).

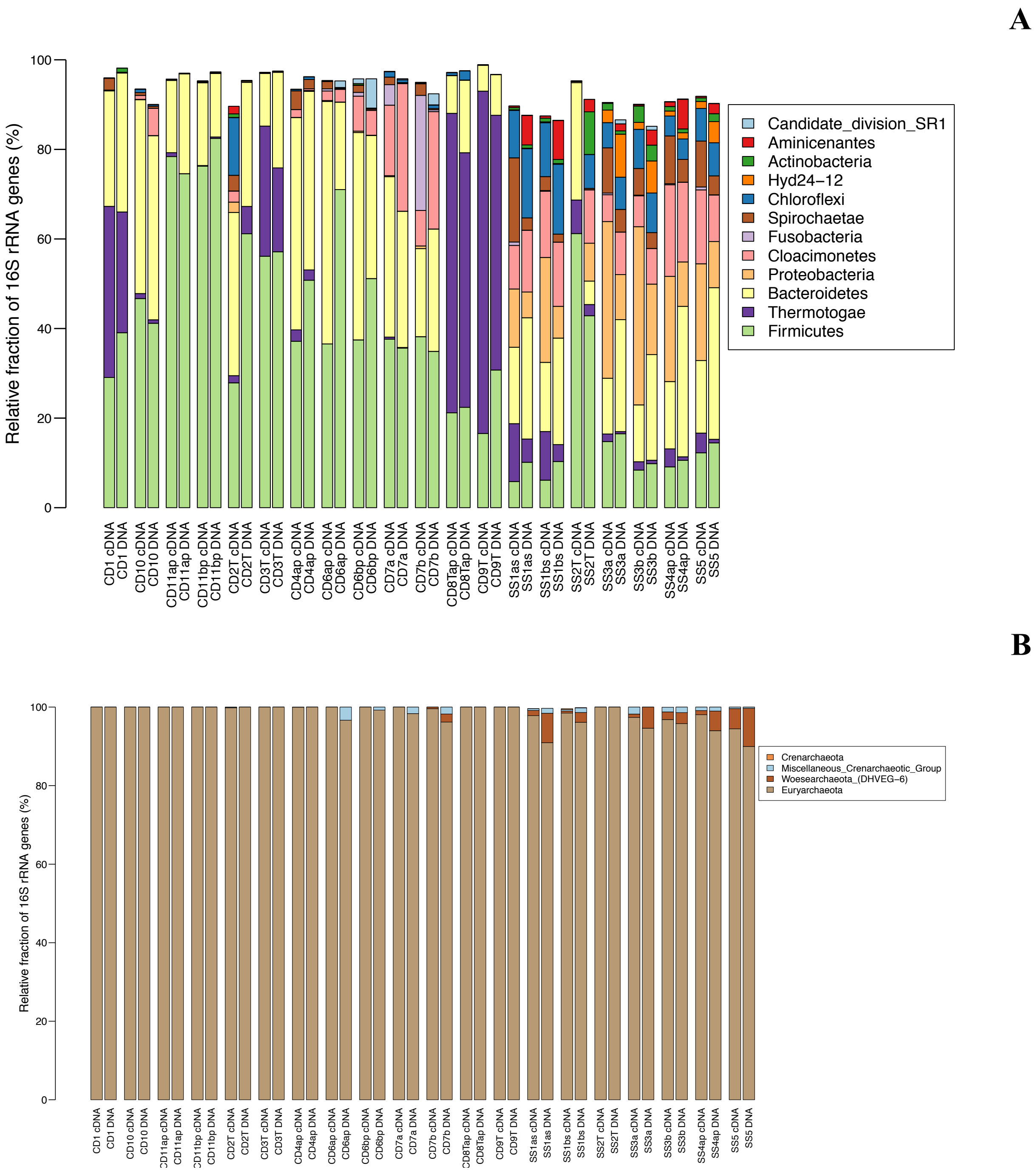

Supplemental Figure S2. Relative sequence fractions of: (A) the 12 most abundant *Bacteria* phyla in the 16S rRNA and rRNA gene profiles of all full-scale digesters; (B) the 4 most abundant *Archaea* phyla in the 16S rRNA and rRNA gene profiles of all full-scale digesters. rRNA fractions are denoted ‘cDNA’. and rDNA fractions are denoted ‘DNA’. Candidate Division SR1 and Hyd24-12 are candidate phyla for which no cultured representatives have been found to date.

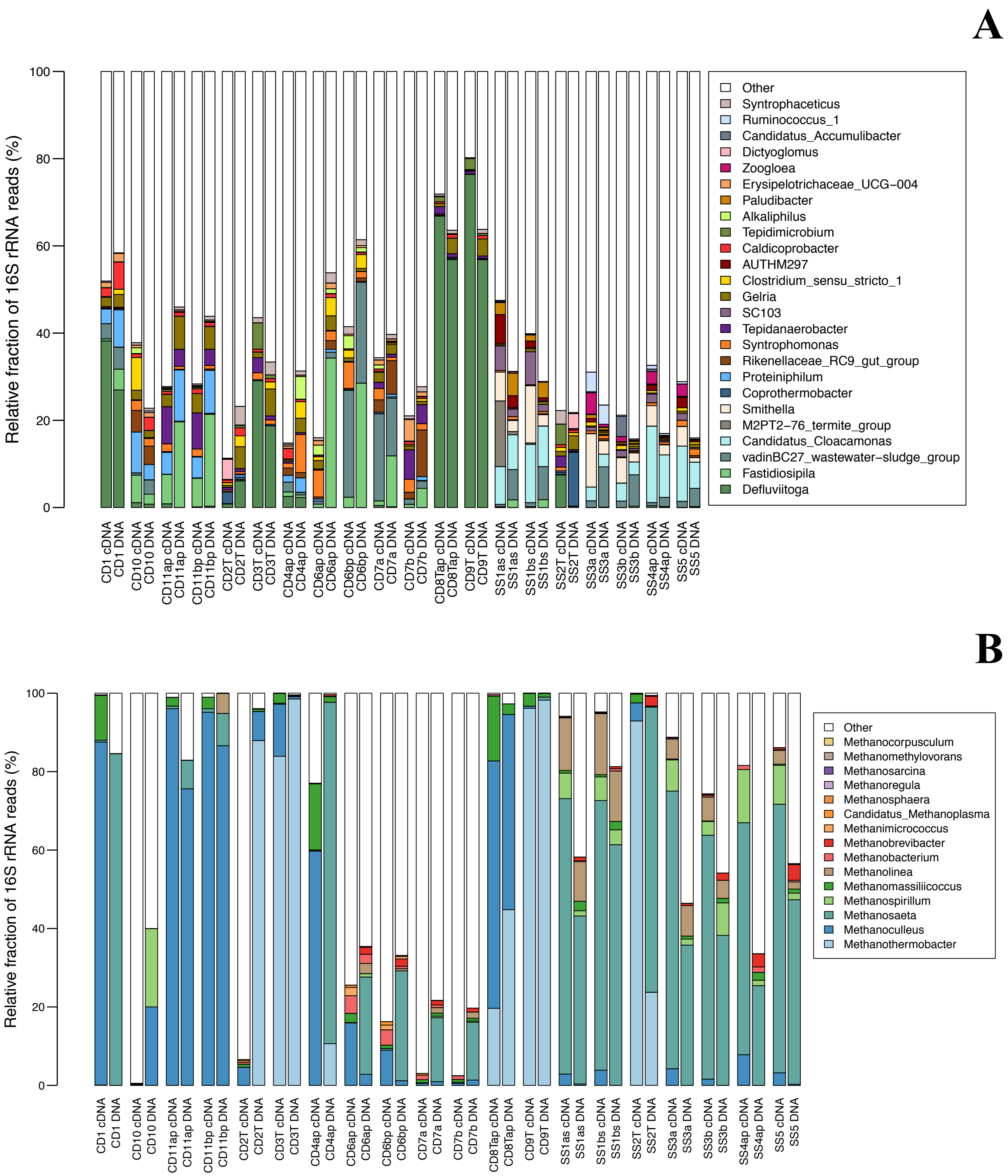

Supplemental Figure S3. Relative sequence fractions of: (A) the 25 most abundant *Bacteria* genera in the 16S rRNA and rRNA gene profiles of all full-scale digesters; (B) the 15 most abundant *Archaea* genera in the 16S rRNA and rRNA gene profiles of all full-scale digesters. rRNA fractions are denoted 'cDNA'. and rDNA fractions are denoted 'DNA'. Read fractions not taxonomically classified within the top 25 most abundant genera are included as 'Other'.

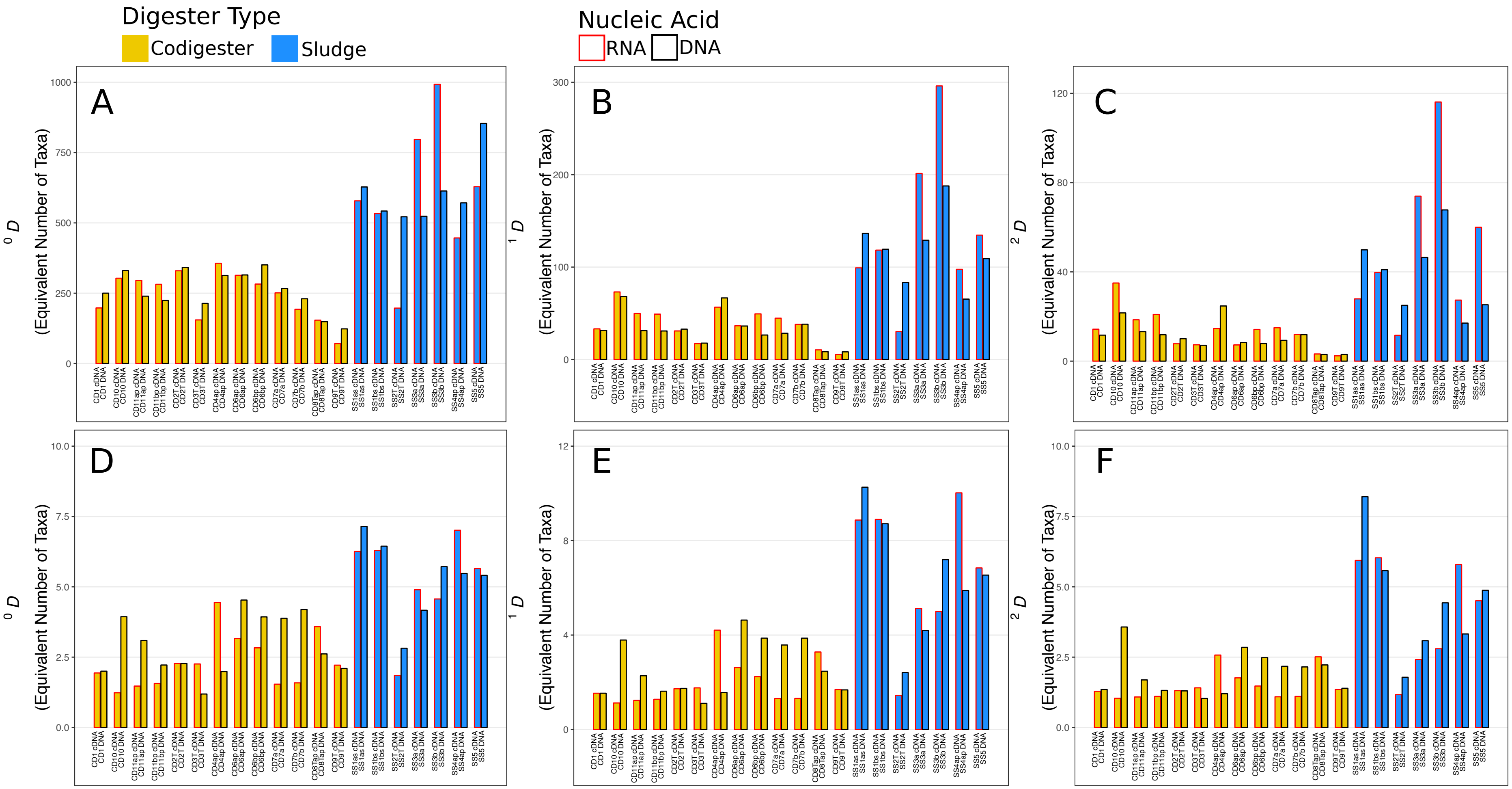

Figure S4. Hill diversity numbers (${}^{q}D$) in 16S rRNA and rDNA profiles for *Bacteria* at orders of (A) $q = 0$. (B) $q = 1$. and (C) $q = 2$. and for *Archaea* at orders of (D) $q = 0$. (E) $q = 1$. and (F) $q = 2$. rRNA samples are denoted 'cDNA'. and rDNA samples are denoted 'DNA'.

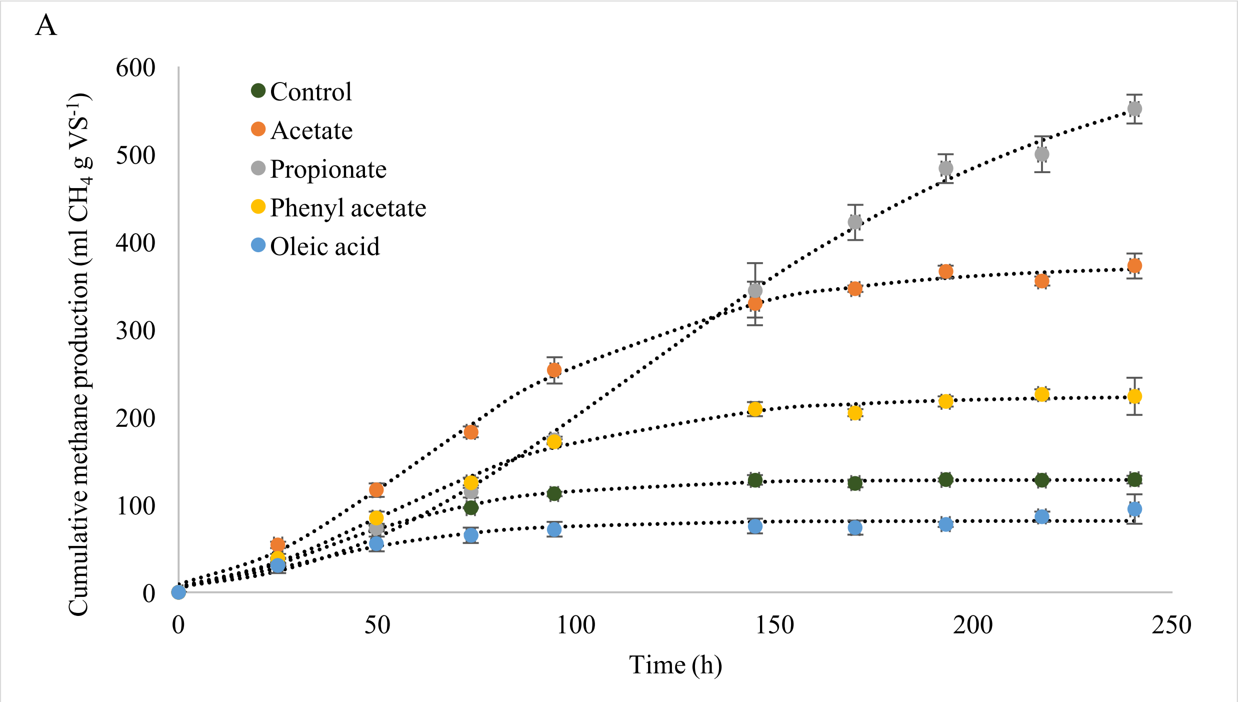

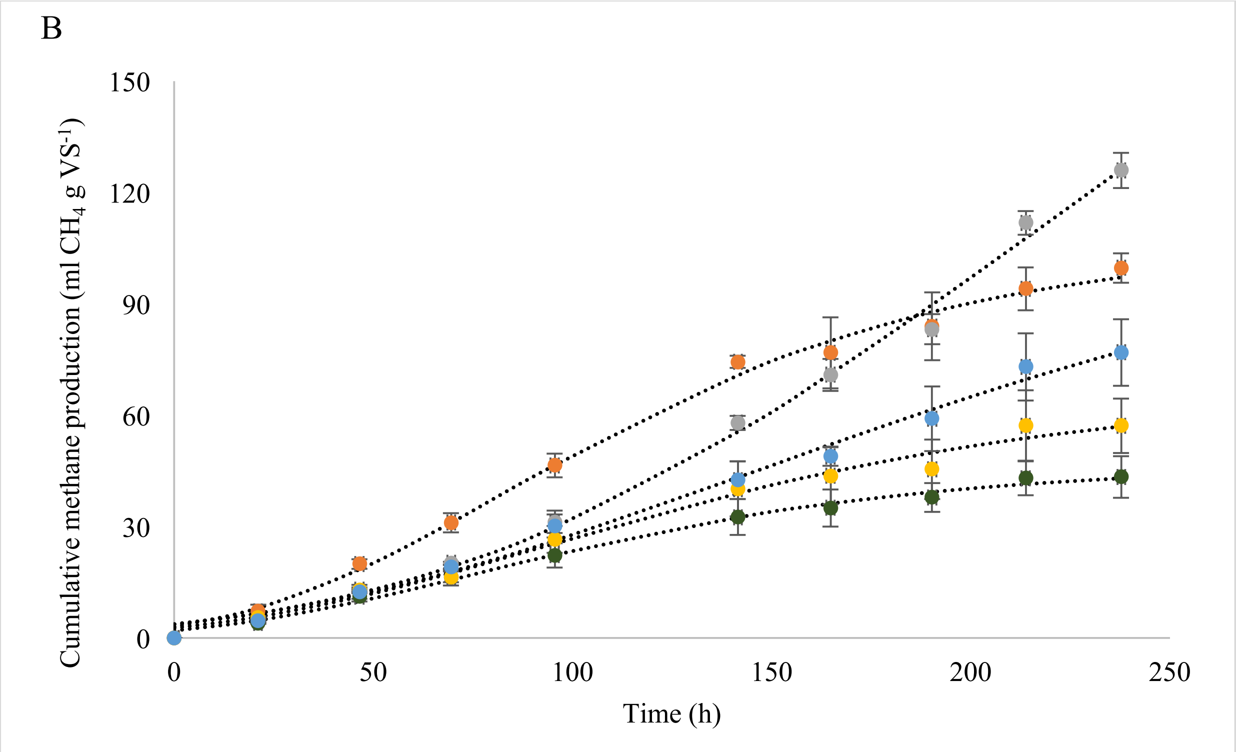

Supplemental Figure S5. Example plots of fitting the Gompertz model (dashed lines) to cumulative methane production in the batch kinetics assays inoculated with sludge samples from SS1bs (A) and CD1 (B) reactors. The error bars indicate standard deviation of methane production in triplicate assays. The goodness-of-fit for all nonlinear regression analyses (r^2) ranged between 0.90 and 0.99.
